# Influence of Oxygen-Containing Sulfur Flavor Molecules on the Stability of β-Carotene under UVA Irradiation

**DOI:** 10.3390/molecules24020318

**Published:** 2019-01-16

**Authors:** Gong-Liang Zhang, Hong-Yan Wu, Ying Liang, Jie Song, Wei-Qi Gan, Hong-Man Hou

**Affiliations:** 1 School of Food Science and Technology, Dalian Polytechnic University, Dalian 116034, China; zgl_mp@163.com (G.-L.Z.); sj101521@163.com (J.S.); 17317138996@163.com (W.-Q.G.); 2 Graduate School of Environmental and Life Science, Okayama University, Okayama 700-8530, Japan; wuhongyan1908@hotmail.com (H.-Y.W.); dlliangying@163.com (Y.L.)

**Keywords:** oxygen-containing sulfur flavor molecules, β-carotene, bis(2-methyl-3-furyl) disulfide (BMFDS), oxidation products

## Abstract

The influence of 11 kinds of oxygen-containing sulfur flavor molecules was examined on β-carotene stability under UVA irradiation in ethanol system. Both the effects of sulfides on dynamic degradation of β-carotene and the relation between structure and effect were investigated. The oxidation products of β-carotene accelerated by sulfides under UVA irradiation were also identified. The results indicated that the disulfides had more obvious accelerative effects on the photodegradation of β-carotene than mono sulfides. The degradation of β-carotene after methyl (2-methyl-3-furyl) disulfide (MMFDS), methyl furfuryl disulfide (MFDS) and bis(2-methyl-3-furyl) disulfide (BMFDS) exposure followed first-order kinetics. Furan-containing sulfides such as MMFDS and BMFDS showed more pronounced accelerative effects than their corresponding isomers. The oxidation products were identified as 13-*cis*-β-carotene, 9,13-di-*cis*-β-carotene and all-*trans*-5,6-epoxy-β-carotene. These results suggest that both the sulfur atom numbers and the furan group in oxygen-containing sulfides play a critical role in the photooxidation of β-carotene.

## 1. Introduction

Carotenoids are natural pigments of the isoprenoid family, commonly biosynthesized in fruits and vegetables [1], presenting potential physiological benefits, such as antioxidants in food and pro-vitamin A activity [2]. As one of the most commonly used carotenoids, β-carotene is expected to be conducive to health because of its valuable nutritional properties and antioxidant capacities, which confer on this compound an important role in lowering the risk of cataracts [3], inhibiting age-related macular degeneration [4], and enhancing the prevention of cardiovascular diseases [5].

However, due to its poor water solubility [6] and low bioavailability [7,8] during food processing and storage, widespread applications of β-carotene in food matrices normally suffer considerable challenges. Moreover, the restriction of β-carotene utilization as a nutritional ingredient in the food industry is currently also attributed to the existence of numerous unsaturated groups, resulting in high vulnerability to degradation reaction when exposed to light, heat and other external factors [9,10]. It has been reported that strong illumination can influence the stability of β-carotene extracted from palm oil, unveiling the formation of cis isomers [11]. The in-depth study carried out by Ayu et al. [12], who investigated interactive influence of tocopherols, tocotrienols, and β-carotene in the process of photooxidation of red palm oil, suggesting that the degradation of β-carotene easily occurs under the irradiation of light. Apart from that, a comparison of β-carotene degradation under different UV stresses was conducted by Chen et al. [13], which showed that the longer the wavelength applied, the faster the degradation rate. 

Several reports have also focused on the effects of chemical substances and their stability. The presence of 1,4-dimethylnaphthalene-1,4-endoperoxide and lycopene had the potential to induce the generation of (9*Z*)-, (13*Z*)- and (15*Z*)-β-carotene, which was associated with the formation of singlet oxygen [14]. Lewis acids, including titanium tetrachloride and ferric chloride, can catalyze the degradation of β-carotene to form an intermediate radical carbocation [15].

Currently, more than 300 sulfides have been registered as Generally Recognized as Safe (GRAS) substances with various threshold limits, making them the critical food flavors [16]. Biological functions including antithrombotic [17], antimicrobial [18], anticancer [19], and anti-inflammatory activities [20] in combination with their attractive odor characteristics such as garlic, onion, meat and nut flavors, have increased their feasibility of acting as food additives. In our previous studies, we discovered that dimethyl sulfides exerted apoptosis-inducing effects in leukemia cell lines via the generation of reactive oxygen species, especially for dimethyl trisulfide(Me_2_S_3_) and dimethyl tetrasulfide(Me_2_S_4_) [21]. Furthermore, β-carotene combined with Me_2_S_4_ under UVA irradiation presented a synergistic action in inhibiting the viability of HL-60 cells viability, and elevating caspase-3 levels [22], mostly like probably raising the possibility of the reaction between sulfides and β-carotene assisted by UVA.

In this study, we selected 11 kinds of oxygen-containing sulfur flavor molecules, commonly used in the food industry, as experimental materials to examine their influence on β-carotene stability under UVA irradiation. Moreover, both the dynamic analysis of β-carotene degradation and the structural effects of sulfides that accelerated the degradation of β-carotene were investigated to provide a clearer and better comprehension of their acceleration effects. Furthermore, the oxidation products of β-carotene under UVA irradiation were also analyzed in order to elucidate its degradation mechanism. 

## 2. Results

### 2.1. The Effects of Oxygen-Containing Sulfur Flavor Molecules on β-Carotene Degradation under UVA Irradiation 

The structures of 11 kinds of oxygen-containing sulfur flavor molecules are shown in Table 1. Most of the sulfides contain furan or furfuryl group, such as 2-methyl-3-(methylthio) furan (MMTF) and methyl furfuryl disulfide (MFDS). Some sulfides contain different numbers of sulfur atoms but have the same side chain groups, such as MMTF and methyl (2-methyl-3-furyl) disulfide (MMFDS), difurfuryl sulfide (DFS) and difurfuryl disulfide (DFDS). Furthermore, it is worth noting the existence of isomers, such as bis(2-methyl-3-furyl) disulfide (BMFDS) and DFDS, MMFDS and MFDS. 

**Figure 1:**
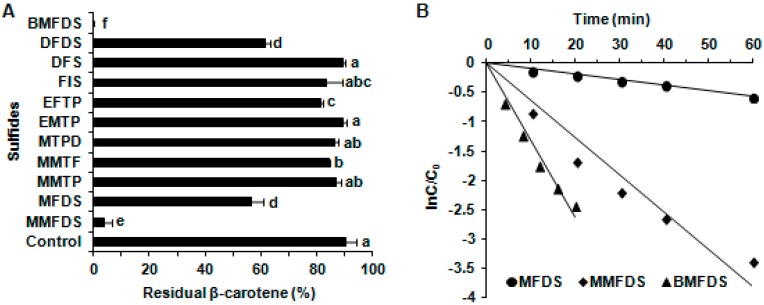
The effect of oxygen-containing sulfides on the degradation of β-carotene in ethanol under UVA irradiation. β-Carotene was treated with various oxygen-containing sulfur flavor molecules under UVA irradiation for 60 min (**A**) or within 60 min (**B**) in ethanol system. The residual β-carotene (**A**) and the first-order kinetics curve of β-carotene degradation (**B**) were determined. The bar results are expressed as means ± SD from three independent replicates. Different small letters show significant differences (*p* < 0.05).

The effects of 11 kinds of oxygen-containing sulfur flavor molecules assisted by UVA irradiation on β-carotene stability are shown in Figure 1A. After irradiating under UVA for 60 min in which the light intensity was 2.5 mW/cm^2^, the contents of β-carotene in all groups showed a reducing trend. To gain more knowledge about the correlation between the structure of coexistent sulfides and the degradation ratios of β-carotene, the remaining amounts of β-carotene were compared according to the structural characteristics of coexistent sulfides. The amounts of β-carotene treated with MMFDS and BMFDS were dramatically decreased by approximately 96.05% and 99.70%, respectively (*p* < 0.05). Likewise, the remaining amount of β-carotene in the presence of MFDS declined approximately 43.64%. These findings proved the fact that natural sulfur substance may affect β-carotene stability.

### 2.2. The Effects of Furan-Containing Sulfides on β-Carotene Degradation under UVA Irradiation

The order of the reaction with respect to the photodegradation of β-carotene was acquired according to Equation (2) to examine the changes of β-carotene concentration with time after UVA irradiation. 

As presented in Figure 1B, β-carotene degradation upon exposure to MMFDS, BMFDS and MFDS followed first-order kinetics, consistent with the kinetic model in dichloromethane system [23]. The corresponding kinetic parameters are listed in Table 2. It can be seen that the presence of BMFDS in ethanol significantly improved the k and shortened the t_1/2_ of β-carotene degradation, compared with the other two sulfides. The k and t_1/2_ were 0.131 min^−1^ and 5.29 min for BMFDS, while they were 0.0633 min^−1^ and 10.95 min for MMFDS and 0.0095 min^−1^ and 72.96 min for MFDS, respectively. Furthermore, it should also be noticed that these three kinds of sulfides (MMFDS, BMFDS and MFDS) having obviously promoting effects on the degradation of β-carotene contain at least two sulfur atoms, which is consistent with the previous report [24]. These results suggest that sulfides with more sulfur atoms might trigger a stronger chemical effect on β-carotene stability in ethanol model system. 

### 2.3. Kinetics of β-Carotene Degradation Treated with BMFDS under UVA Irradiation 

To further clarify the acceleration effect of BMFDS, the degradation kinetics parameters of β-carotene under UVA irradiation were determined. As shown in Table 3, the photodegradation of β-carotene treated by BMFDS in an ethanol system followed first-order kinetics. In terms of the rate constant k, the degradation degree of β-carotene treated with BMFDS was much higher, approximately 156 times than that in the control group. Therefore, our findings demonstrated that β-carotene degradation was followed first-order kinetics after treatment with BMFDS. 

### 2.4. The Analysis of Photooxidation Products of β-Carotene Treated with BMFDS under UVA Irradiation 

**Figure 2:**
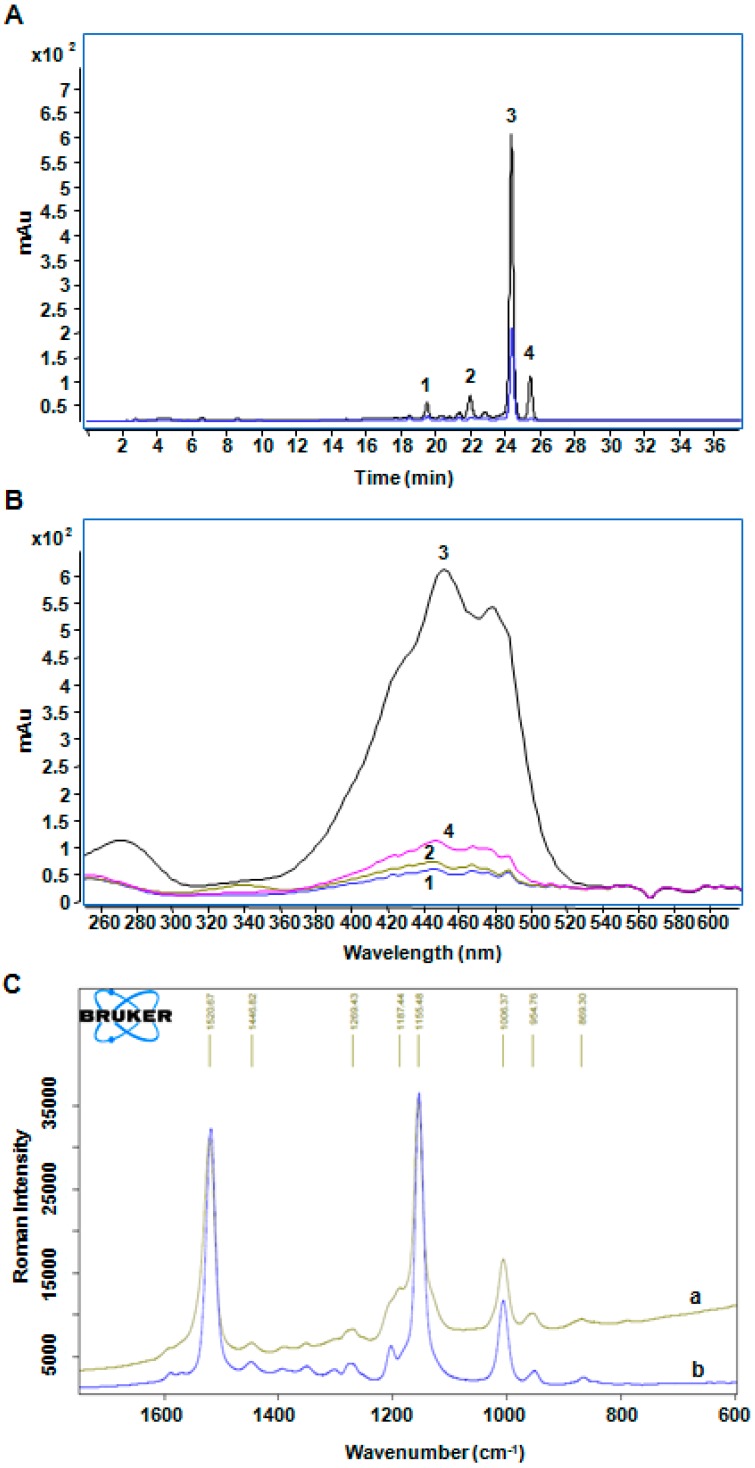
The products of β-carotene treated with BMFDS under UVA irradiation. HPLC chromatograms (**A**) and UV-Vis spectra (**B**) of β-carotene oxidation products induced by BMFDS treatment for 5 min in ethanol under UVA irradiation. The blue line in HPLC chromatograms is referred to standard β-carotene. Peak identification for (**A**) and (**B**): Peak 1, all-*trans*-5,6-expoxy-β-carotene; Peak 2, 13-*cis*-β-carotene; Peak 3, all-*trans*-β-carotene; Peak 4, 9,13-di-*cis*-β-carotene. Raman spectra (**C**) at different wave number corresponded to β-carotene under UVA irradiation for 5 min with BMFDS (**a**) and without BMFDS (**b**), respectively.

**Figure 3:**
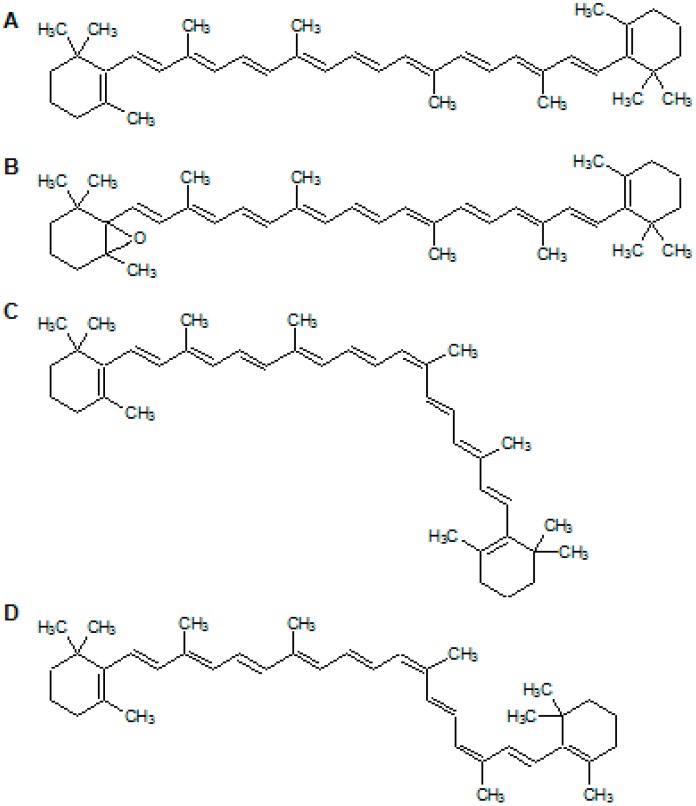
The structure of β-carotene and its oxidation products induced by BMFDS under UVA irradiation. (**A**) all-*trans*-β-carotene; (**B**) all-*trans*-5,6-epoxy-β-carotene; (**C**) 13-*cis*-β-carotene; (**D**) 9,13-di-*cis*-β-carotene.

According to the remarkable acceleration effect of BMFDS, HPLC-DAD-APCI-MS combined with Raman spectroscopy was applied to make a preliminary identification about the oxidation products of β-carotene, given their low contents and rather complex process of products collection. The chromatographic and spectral data of the oxidation products of β-carotene by HPLC-DAD-ACPI-MS are shown in Figure 2 and Table 4. The chromatographic peaks of leading β-carotene oxidation products had the same peak time in both the experimental group and the control group. Therefore, it was supposed that β-carotene treated with or without BMFDS under UVA irradiation had the same oxidation products (Figure 2A). In comparison to the retention time of a standard product (24.322 min) (Table 4), peak 3 was confirmed as all-*trans*-β-carotene (24.319 min) (Figure 3A). The UV spectra data are depicted in Figure 2B, and among the four obvious peaks, peak 2 and peak 4 were identified tentatively as 13-*cis*-β-carotene [25] and 9,13-di-*cis*-β-carotene [26], respectively (Figure 3C,D) according to the spectral characteristics and Q-ratios (Table 4), which were stipulated as the absorbance ratio of the middle main absorption peak to the cis peak. 

The Raman spectra at various wavenumber (cm^−1^) of β-carotene in ethanol system treated with or without BMFDS under UVA irradiation are displayed in Figure 2C. By comparison of Figure 2C (a) and Figure 2C (b), both υ1 (–C=C–) at 1520.67 cm^−1^ and υ2 (–C=C–C=C–) at 1155.48 cm^−1^ can be observed. However, the distinction existed in the appearance of a new polar function group (C–O) presented in Figure 2C (a). Combined with its *m*/*z* of 553 (Table 4), this new vibration peak was identified tentatively as all-*trans*-5,6-epoxy-β-carotene (Figure 3B). As would have been expected, mono-epoxide is susceptible to generate from different sorts of carotenoids.

## 3. Discussion

β-Carotene was susceptible to be affected when exposed to external factors, consistent with previous reports, which considered that light can exert influence on β-carotene degradation [27,28,29]. In this study, sulfides have also showed to be involved in interfering the stability of β-carotene, which agreed with the report of Wei et al., implying that the stability of β-carotene can be significantly influenced when chitosan-(−)-epigallocatechin-3-gallate conjugates on β-carotene emulsions covered by sodium caseinate [30]. Moreover, in agreement with previous studies, both the number of sulfur atoms and the type of side group can affect the accelerated degradation of β-carotene under UVA irradiation [24,31]. It has been reported that the coexistence of disulfides can remarkably decrease the residual ratios of β-carotene to approximately 51.8–69.1%, while the presence of mono sulfides did not show obvious accelerating effects compared to the absence of mono sulfides [24]. 

β-Carotene degradation upon exposure to MMFDS, BMFDS and MFDS followed first-order kinetics, consistent with the kinetic model in dichloromethane system [23]. On the basis of the previous report which focused on the phenomenon of the existence of isomers [32], we also studied the accelerated effects of side groups among these sulfides on the degradation of β-carotene. Although MMFDS and MFDS both possess the same molecular formula (C_6_H_8_OS_2_), MMFDS showed a stronger accelerated degradation effect than MFDS. It is presumably because there is a methyl group and a furan group on the side of the disulfide bond in MMFDS, while a methyl group and a furfuryl group exist on the side of the disulfide bond in MFDS. For DFDS and its corresponding isomer BMFDS. Similarly, there is a furan group on both ends of the disulfide bond in BMFDS, while there is a furfuryl group on each side of the disulfide bond in DFDS. Their different abilities to promote the degradation of β-carotene can be related to the existence of various side groups. These results may account for the fact that furan-containing sulfur flavor molecules (MMFDS and BMFDS) showed a much more remarkable acceleration effect on the degradation of β-carotene than furfuryl-containing sulfur flavor molecules (MFDS and DFDS, respectively). Therefore, the number of sulfur atoms and the furan group in oxygen-containing sulfur flavor molecules may play a critical role in the accelerated degradation of β-carotene under UVA irradiation in ethanol system.

Several studies have investigated the order of kinetics on the degradation of β-carotene in different model systems under various conditions. It also followed a first order reaction under ambient storage, ultraviolet radiation and even heat treatments [13]. The photodegradation of β-carotene treated by BMFDS in ethanol system followed first-order kinetics, which agreed with previous studies carried out in food model systems such as carrots [33], oil/carrot emulsion system [34], oil model systems [10,35] and pulp or juices [36,37]. Ferreira et al. observed a first-order reaction for β-carotene degradation in a low-moisture and aqueous model system, as well as in lyophilized guava under different processing and storage conditions [38].

In addition, our results were in good agreement with Li et al. who summarized that the *trans*-*cis* isomerization of carotenoids can be generated via contacting with acids, thermal treatment or light [39]. It has been a long time since 13-*cis*-β-carotene was recognized as one of the main *cis* forms of β-carotene in food [40]. Chen et al. even analyzed it by different processing means, including over-heating and (non)-iodine-catalyzed photodegradation [41]. In addition, 9,13-di-*cis*-β-carotene was also confirmed as a common β-carotene degradation product according to Glaser et al. [42]. Moreover, our founding was consistent with Handelman et al., who had detected 5,6-epoxide of β-carotene through utilizing HPLC with mass analysis [43]. Similarly, Zeb identified all-*trans*-5,6-epoxy-β-carotene by an HPLC system and single ion monitoring mass spectrometry as well [44].

## 4. Materials and Methods 

### 4.1. Materials and Chemicals 

Eleven kinds of oxygen-containing sulfur flavors and β-carotene were obtained from Sigma-Aldrich (St. Louis, MO, USA). The structures of these sulfides are presented in Table 1. Methanol and methyl *tert*-butyl ether (MTBE)were procured from Damao Chemical factory (Tianjin, China) and Fisher Scientific (Pittsburgh, PA, USA), respectively. The other chemicals and reagents were of analytical grade. 

### 4.2. Preparation of the Model Systems

For the preparation of the ethanol model system, 1 mg β-carotene was dissolved in 15 mL ethanol according to Onsekizoglu et al. [27] with minor modifications. The β-carotene solution was prepared daily and kept in the dark at 4 °C before use. Stock solutions of 11 kinds of sulfides were prepared in ethanol at a concentration of 10 mM and kept at 4 °C prior to use.

### 4.3. Kinetic Analysis of β-Carotene Degradation

The working solutions of β-carotene were transferred into quartz cuvettes, followed by the addition of 10 μL sulfur flavors. The control was performed with 10 μL ethanol. Then, the mixture was treated by UVA light (2.5 mW/cm^2^) with the aim of assessing the degradation kinetics of β-carotene treated with sulfide. The degradation of β-carotene was measured immediately in a UV-1750 spectrophotometer (Shimadzu, Tokyo, Japan) at the wavelength of 450 nm for 60 min, which was monitored every 10 min. All measurements were performed in triplicate and data are expressed as mean of three independent experiments.

### 4.4. Degradation Kinetics Modeling

The trial-and-error procedure was carried out in accordance with the integral method outlined by Sánchezet al. [45] to determine the reaction order of theβ-carotene degradation. Different order models can be represented as follows:

*c* − *c*_0_ = −*kt* (1)

ln *c*/*c*_0_ = −*kt* (2)

1/*c* − 1/*c*_0_ = *kt* (3)

In these formulas, *c* (μM) is thereactantconcentrationat a given time, *c*_0_ (μM) is the initial reactant concentration, *k* (min^−1^) is the degradation rate constant, and *t* (min) is the treatment time.

### 4.5. Analysis of β-Carotene Treated with UVA Irradiation and BMFDS

The working solutions of β-carotene were transferred into quartz cuvettes, followed by the addition of 10 μL BMFDS. The control was performed with 10 μL ethanol. Then, the mixture containing β-carotene and BMFDS was placed under a UV lamp (Shimadzu, Japan) with an intensity of 2.5 mW/cm^2^ for 5 min, followed by drying completely under a nitrogen stream. The residue was redissolved in 0.1 mL MTBE before use.

The further analysis was carried out and relative parameters were applied according to Santos et al. [46]. Briefly, once redissolved, the solution was passed through a 0.22 μm filter, followed by the injection into an HPLC-DAD-APCI-MS system (Agilent, Santa Clara, CA, USA) for closer analysis. A YMC C_30_ column (250 × 4.6 mm, 5 μm) and gradient mobile phase of methanol-MTBE-water (85:15:5, *v*/*v*/*v*) and MTBE (100%) were used for β-carotene detection.

### 4.6. Determination of Degradation Products by Raman Spectroscopy

The Raman spectra of the degradation products were recorded on a Raman spectrometer (Bruker Instruments Inc., Bill-erica, MA, USA). The wave number was in the range of 400–4000 cm^−1^ using the 785 nm as the excitation line. The power was 10 Mw while the integration time was 20 s.

### 4.7. Statistical Analysis

All the data were expressed as the mean ± SD or mean and subjected to the Student’s *t*-test for statistical analysis. Statistical significance was considered at a *p* < 0.05.

## 5. Conclusions

Overall, through applying the results we obtained and drawing upon the information provided by other studies, it is supposed that the oxidation products of β-carotene treated with BMFDS under UVA irradiation in ethanol system might include 13-*cis*-β-carotene, 9,13-di-*cis*-β-carotene and all-*trans*-5,6-epoxy-β-carotene. Our results might shed new light on the accelerative effect of BMFDS on the photodegradation of β-carotene. More insights into the mechanism involved in degradation of oxidation products of BMFDS-treated β-carotene should be further studied further.

## Figures and Tables

**Table 1:** Structure of eleven oxygen-containing sulfur flavor molecules.

FEMA Number	Name	Abbreviation	Structure
3573	Methyl (2-Methyl-3-furyl) disulfide	MMFDS	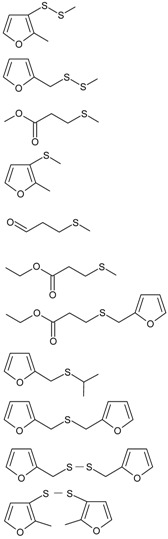
3362	Methyl furfuryl disulfide	MFDS	
2720	Methyl 3-methylthiopropionate	MMTP	
3949	2-Methyl-3-(methylthio) furan	MMTF	
2747	3-(Methylthio) propionaldehyde	MTPD	
3343	Ethyl 3-methylthiopropionate	EMTP	
3674	Ethyl 3-(furfurylthio) propionate	EFTP	
3161	Furfuryl isopropyl sulfide	FIS	
3238	Difurfuryl sulfide	DFS	
3146	Difurfuryl disulfide	DFDS	
3259	Bis (2-methyl-3-furyl) disulfide	BMFDS	

FEMA: Flavor and Extract Manufacturers Association of the United States.

**Table 2:** Degradation kinetics parameters of β-carotene in the presence of BMFDS, MMFDS, MFDS under UVA irradiation in ethanol system.

Sulfides	K (min^−1^)	R^2^	t_1/2_ (min)
MFDS	0.0095	0.9959	72.96
MMFDS	0.0633	0.9356	10.95
BMFDS	0.131	0.9720	5.29

BMFDS: Bis (2-methyl-3-furyl) disulfide, MMFDS: Methyl (2-Methyl-3-furyl) disulfide, MFDS: Methyl furfuryl disulfide.

**Table 3:** Degradation kinetics parametersβ-carotene in the presence of BMFDS under UVA irradiation.

	R Zero-Order (c)	R First-Order (ln c)	R Second-Order (1/c)	k (min^−1^)	R^2^
Control	0.9468	0.9512	0.9554	0.0012	0.9785
BMFDS	0.6376	0.9766	0.9141	0.1879	0.9537

c is the concentration of reactant.

**Table 4:** Product identification of β-carotene treated with BMFDS under UVA irradiation.

Peak	Retention Time (min)	Molecular Ion, Formula	Isomer	λ (nm)		Q-Ratio	
				Found	Reported	Found	Reported
1	19.467	553, C_18_H_57_O	All-*trans*-5,6-expoxy-β-carotene	446	444		
2	21.953	537, C_18_H_57_	13-*cis*-β-carotene	340 446	340 445	0.41	0.41
3	24.319	537, C_18_H_57_	All-*trans*-β-carotene	451	455		
4	25.399	537, C_18_H_57_	9,13-di-*cis*-β-carotene	350 446	335 439	0.16	0.16

## References

[1] Durante M., Lenucci M.S., D’Amico L., Piro G., Mita G. (2014). Effect of drying and co-matrix addition on the yield and quality of supercritical CO_2_ extracted pumpkin (*Cucurbita moschata* Duch.) oil. Food Chem..

[2] Lemmens L., Colle I., Buggenhout S.V., Palmero P., Loey A.V., Hendrickx M. (2014). Carotenoid bioaccessibility in fruit-and vegetable-based food products as affected by product (micro) structural characteristics and the presence of lipids: A review. Trends Food Sci. Technol..

[3] Hou Z.Q., Liu Y.W., Lei F., Gao Y.X. (2014). Investigation into the in vitro release properties of β-carotene in emulsions stabilized by different emulsifiers. LWT Food Sci. Technol..

[4] Gul K., Tak A., Singh A.K., Singh P., Yousuf B., Wani A.A. (2015). Chemistry, encapsulation, and health benefits of β-carotene—A review. Cogent. Food Agric..

[5] Tanaka T., Shnimizu M., Moriwaki H. (2012). Cancer chemoprevention by carotenoids. Molecules.

[6] Huang Q.R., Yu H.L., Ru Q.M. (2010). Bioavailability and delivery of nutraceuticals using nanotechnology. J. Food Sci..

[7] Salvia-Trujillo L., Qian C., Martín-Belloso O., McClements D.J. (2013). Influence of particle size on lipid digestion and beta-carotene bioaccessibility in emulsions and nanoemulsions. Food Chem..

[8] Salvia-Trujillo L., Qian C., Martin-Belloso O., McClements D.J. (2013). Modulating β-carotene bioaccessibility by controlling oil composition and concentration in edible nanoemulsions. Food Chem..

[9] Boon C.S., Mcclements D.J., Weiss J., Decker E.A. (2010). Factors influencing the chemical stability of carotenoids in foods. Crit. Rev. Food Sci..

[10] Achir N., Randrianatoandro V.A., Bohuon P., Laffargue A., Avallone S. (2010). Kinetic study of β-carotene and lutein degradation in oils during heat treatment. Eur. J. Lipid Sci. Technol..

[11] Guan Y., Wu J., Zhong Q. (2016). Eugenol improves physical and chemical stabilities of nanoemulsions loaded with β-carotene. Food Chem..

[12] Ayu D.F., Andarwulan N., Hariyadi P., Purnomo E.H. (2016). Effectof tocopherols, tocotrienols, β-carotene and chlorophyll on the photo-oxidative stability of red palm oil. Food Sci. Biotechnol..

[13] Chen H.Q., Zhong Q.X. (2015). Thermal and UV stability of β-carotene dissolved in peppermint oil microemulsified by sunflower lecithin and Tween 20 blend. Food Chem..

[14] Heymann T., Heinz P., Glomb M.A. (2015). Lycopene inhibits the isomerization of β-carotene during quenching of singlet oxygen and free radicals. J. Agric. Food Chem..

[15] Rajendran V., Chen B.H. (2007). Isomerization of β-carotene by titanium tetrachloride catalyst. J. Chem. Sci..

[16] Liu Y.P., Chen H.T., Yin D.C., Sun B.G. (2010). Synthesis and odor evaluation of five new sulfur-containing ester flavor compounds from 4-ethyloctanoic acid. Molecules.

[17] Apitz-Castro R., Badimon J.J., Badimon L. (1992). Effect of ajoene, the major antiplatelet compound from garlic, on platelet thrombus formation. Thromb. Res..

[18] Naganawa R., Iwata N., Ishikawa K., Fukuda H., Fujino T., Suzuki A. (1996). Inhibition of microbial growth by ajoene, a sulfur-containing compound derived from garlic. Appl. Environ. Microb..

[19] Taylor P., Noriega R., Farah C., Abad M.J., Arsenak M., Apitz R. (2006). Ajoene inhibits both primary tumor growth and metastasis of B16/BL6 melanoma cells in C57BL/6 mice. Cancer Lett..

[20] Dirsch V.M., Vollmar A.M. (2001). Ajoene, a natural product with nonsteroidal anti-inflammatory drug (NSAID)-like properties. Biochem. Pharmacol..

[21] Zhang G.L., Wu H.T., Zhu B.W., Shimoishi Y., Nakamura Y., Murata Y. (2008). Effect of dimethyl sulfides on the induction of apoptosis in human leukemia Jurkat cells and HL-60 cells. Biosci. Biotechnol. Biochem..

[22] Zhang G.L., Wu H.T., Zhu B.W., Shimoishi Y., Nakamura Y., Murata Y. (2009). Induction of apoptosis by beta-carotene and dimethyl tetrasulfide assisted by UVA irradiation in HL-60 cells. Biosci. Biotechnol. Biochem..

[23] Sun Y., Ma G., Ye X., Kakuda Y., Meng R. (2010). Stability of all-trans-beta-carotene under ultrasound treatment in a model system: Effects of different factors, kinetics and newly formed compounds. Ultrason. Sonochem..

[24] Arita S., Ando S., Hosoda H., Sakaue K., Nagata T., Murata Y., Shimoishi Y., Tada M. (2005). Acceleration effect of sulfides on photodegradation of carotenoids by UVA irradiation. Biosci. Biotechnol. Biochem..

[25] Lowe G.M., Vlismas K., Graham D.L., Carail M., Caris-Veyrat C., Young A.J. (2009). The degradation of (all-*E*)-β-carotene by cigarette smoke. Free Radic. Res..

[26] Dan Q., Chen Z.R., Li H.R. (2009). Effect of heating on solidβ-carotene. Food Chem..

[27] Onsekizoglu P., Gökmen V., Acar J. (2005). Degradation of β-carotene with the effects of light and sulfur dioxide may be responsible for the formation of white spot in dried apricots. Eur. Food. Res. Technol..

[28] Spada J.C., Noreña C.P.Z., Marczak L.D.F., Tessaro I.C. (2012). Study on the stability of β-carotene microencapsulated with pinhão (*Araucaria angustifolia* seeds) starch. Carbohydr. Polym..

[29] Chen B.H., Chen T.M., Chien J.T. (1994). Kinetic model for studying the isomerization of alpha- and beta-carotene during heating and illumination. J. Agric. Food Chem..

[30] Wei Z.H., Gao Y.X. (2016). Physicochemical properties of β-carotene bilayer emulsions coated by milk proteins and chitosan–EGCG conjugates. Food Hydrocoll..

[31] Zhang G.L., Zhu B.W., Nakamura Y., Shimoishi Y., Murata Y. (2008). Structure-dependent photodegradation of carotenoids accelerated by dimethyl tetrasulfide under UVA irradiation. Biosci. Biotechnol. Biochem..

[32] Zhang G.L., Liang Y., Zhu J.Y., Jia Q., Gan W.Q., Sun L.M., Hou H.M. (2015). Oxidative stress-mediated antiproliferative effects of furan-containing sulfur flavors in human leukemia Jurkat cells. Food Chem..

[33] Lavelli V., Zanoni B., Zaniboni A. (2007). Effect of water activity on carotenoid degradation in dehydrated carrots. Food Chem..

[34] Knockaert G., Pulissery S.K., Lemmens L., Van B.S., Hendrickx M., Van L.A. (2012). Carrot beta-carotene degradation and isomerization kinetics during thermal processing in the presence of oil. J. Agric. Food Chem..

[35] Aparicio-Ruiz R., Mínguez-Mosquera M.I., Gandul-Rojas B. (2011). Thermal degradation kinetics of lutein, β-carotene and β-cryptoxanthin in virgin olive oils. J. Food Compos. Anal..

[36] Ahmed J., Shivhare U.S., Sandhu K.S. (2002). Thermal degradation kinetics of carotenoids and visual color of papaya puree. J. Food Sci..

[37] Saxena A., Maity T., Raju P.S., Bawa A.S. (2012). Degradation kinetics of colour and total carotenoids in jackfruit (*Artocarpus heterophyllus*) bulb slices during hot air drying. Food Bioprocess Technol..

[38] Ferreira J.E.M., Rodriguez-Amaya D.B. (2008). Degradation of lycopene and β-carotene in model systems and in lyophilized guava during ambient storage: Kinetics, structure, and matrix effects. J. Food Sci..

[39] Li D.J., Xiao Y.D., Zhang Z.Y., Liu C. (2015). Light-induced oxidation and isomerization of all-*trans*-β-cryptoxanthin in a model system. J. Photochem. Photobiol. B.

[40] Chen B.H., Chen Y.Y. (1993). Stability of chlorophylls and carotenoids in sweet potato leaves during microwave cooking. J. Agric. Food Chem..

[41] Chen B.H., Huang J.H. (1998). Degradation and isomerization of chlorophyll a and β-carotene as affected by various heating and illumination treatments. Food Chem..

[42] Glaser T., Lienau A., Zeeb D., Krucker M., Dachtler M., Albert K. (2003). Qualitative and quantitative determination of carotenoid stereoisomers in a variety of spinach samples by use of MSPD before HPLC-UV, HPLC-APCI-MS, and HPLC-NMR online coupling. Chromatographia.

[43] Handelman G.J., Kuijk F.J.G.M.V., Chatterjee A., Krinsky N.I. (1991). Characterization of products formed during the autoxidation of β-carotene. Free Radic. Biol. Med..

[44] Zeb A. (2012). Oxidation and formation of oxidation products of β-carotene at boiling temperature. Chem. Phys. Lipids.

[45] Sánchez A.M., Carmona M., Ordoudi S.A., Tsimidou M.Z., Alonso G.L. (2008). Kinetics of individual crocetin ester degradation in aqueous extracts of saffron (*Crocus sativus* L.) upon thermal treatment in the dark. J. Agric. Food Chem..

[46] Santos J., Herrero M., Mendiola J.A., Oliva-Teles M.T., Ibáñez E., Delerue-Matos C., Oliveira M.B.P.P. (2014). Assessment of nutritional and metabolic profiles of pea shoots: The new ready-to-eat baby-leaf vegetable. Food Res. Int..

